# Predictive Criteria to Study the Pathogenesis of Malaria-Associated ALI/ARDS in Mice

**DOI:** 10.1155/2014/872464

**Published:** 2014-09-02

**Authors:** Luana S. Ortolan, Michelle K. Sercundes, Renato Barboza, Daniela Debone, Oscar Murillo, Stefano C. F. Hagen, Momtchilo Russo, Maria Regina D' Império Lima, José M. Alvarez, Marcos Amaku, Claudio R. F. Marinho, Sabrina Epiphanio

**Affiliations:** ^1^Departamento de Imunologia, Instituto de Ciências Biomédicas, Universidade de São Paulo, Edifício Biomédicas IV, Avenida Professor Lineu Prestes, No. 1730, 05508-900 São Paulo, SP, Brazil; ^2^Departamento de Ciências Biológicas, Universidade Federal de São Paulo, Rua Professor Artur Riedel, No. 275, Jardim Eldorado, 09972-270 Diadema, SP, Brazil; ^3^Instituto de Medicina Tropical de São Paulo, Universidade de São Paulo, Avenida Dr. Enéas Carvalho de Aguiar, No. 470, 05403-000 São Paulo, SP, Brazil; ^4^Departamento de Ciências Exatas e da Terra, Universidade Federal de São Paulo, Rua Professor Artur Riedel, No. 275, Jardim Eldorado, 09972-270 Diadema, SP, Brazil; ^5^Departamento de Parasitologia, Instituto de Ciências Biomédicas, Universidade de São Paulo, Avenida Professor Lineu Prestes, No. 1374, Edifício Biomédicas II Cidade Universitária “Armando Salles Oliveira”, 05508-000 São Paulo, SP, Brazil; ^6^Departamento de Cirurgia, Faculdade de Medicina Veterinária e Zootecnia da Universidade de São Paulo, Avenida Professor Dr. Orlando Marques de Paiva, No. 87, Cidade Universitária, 05508 270 São Paulo, SP, Brazil; ^7^Departamento de Medicina Veterinária Preventiva e Saúde Animal, Faculdade de Medicina Veterinária e Zootecnia da Universidade de São Paulo, Avenida Professor Dr. Orlando Marques de Paiva, No. 87, Cidade Universitária, 05508 270 São Paulo, SP, Brazil; ^8^Departamento de Análises Clínicas e Toxicológicas, Faculdade de Ciências Farmacêuticas, Universidade de São Paulo, Avenida Professor Lineu Prestes, No. 580, Bloco 17, Cidade Universitária “Armando Salles Oliveira”, 05508-000 São Paulo, SP, Brazil

## Abstract

Malaria-associated acute lung injury/acute respiratory distress syndrome (ALI/ARDS) often results in morbidity and mortality. Murine models to study malaria-associated ALI/ARDS have been described; we still lack a method of distinguishing which mice will develop ALI/ARDS before death. This work aimed to characterize malaria-associated ALI/ARDS in a murine model and to demonstrate the first method to predict whether mice are suffering from ALI/ARDS before death. DBA/2 mice infected with *Plasmodium berghei* ANKA developing ALI/ARDS or hyperparasitemia (HP) were compared using histopathology, PaO_2_ measurement, pulmonary X-ray, breathing capacity, lung permeability, and serum vascular endothelial growth factor (VEGF) levels according to either the day of death or the suggested predictive criteria. We proposed a model to predict malaria-associated ALI/ARDS using breathing patterns (enhanced pause and frequency respiration) and parasitemia as predictive criteria from mice whose cause of death was known to retrospectively diagnose the sacrificed mice as likely to die of ALI/ARDS as early as 7 days after infection. Using this method, we showed increased VEGF levels and increased lung permeability in mice predicted to die of ALI/ARDS. This proposed method for accurately identifying mice suffering from ALI/ARDS before death will enable the use of this model to study the pathogenesis of this disease.

## 1. **Introduction**

Malaria is an infectious disease with a huge impact on public health and a high mortality rate. According to the World Health Organization, approximately 3.3 billion people were at risk of contracting malaria in 2011 [1–3]. In some individuals,* Plasmodium* infection may result in severe malaria that can lead to ALI/ARDS [4, 5]. Patients infected with* P. falciparum*,* P. vivax*, and* P. knowlesi* can develop ALI or ARDS with mortality rates of approximately 80% [6, 7]. Malaria-associated ALI/ARDS is thought to be due, in part, to increased alveolar permeability, parasite sequestration, and host immune response; however, the mechanisms behind it are largely unknown [4].

ALI/ARDS can occur at any time during an infection, even after treatment with antimalarial drugs when parasitemia has been reduced (reviewed in [4]). The development of ALI/ARDS, along with its negative outcomes, makes the prospective identification and effective treatment of those who develop this syndrome very important. Though, there is little information on malaria-associated ALI/ARDS progression, resulting in a lack of knowledge of the mechanisms of pathogenesis; therefore, the understanding of mouse models is essential.

Several reports have observed lung injury in mice infected with* P. berghei* (*Pb*) strains [8–19]. The observations have highlighted possible roles for many factors, including platelet-activating factor receptor [8], urokinase receptor [9], ICAM-1 [10, 11], CD40 [12], neutrophils [13], vascular endothelial growth factor (VEGF) [14], epithelium sodium channel activity [15], CD36-dependent parasite sequestration [16], hemozoin deposition [17], and CD8+ T lymphocytes [18], in malaria-associated lung injury.

Recently, there have been many models described that focus on the pulmonary pathology associated with malaria, including the classical C57BL/6 susceptible mouse model of cerebral malaria infection with* Pb*ANKA [16, 19]. Other models have used different parasite/mouse combinations that result in the mice surviving for longer periods of time (without signs of cerebral malaria), theoretically allowing the investigation of disease progression over time [14, 15, 18]. However, none of them were able to identify ALI/ARDS before death. Here, we have characterized a murine model of malaria-associated ALI/ARDS that shows similarities between humans and murine ALI/ARDS. Moreover, we proposed a method for classifying mice suffering from ALI/ARDS before the time of death as a predictive model for malaria-associated ALI/ARDS.

## 2. **Materials and Methods**

### 2.1. Mice and Parasites

DBA/2 male mice 6–10 weeks old (purchased from the Department of Parasitology, University of São Paulo, Brazil) were infected with 1 × 10^6^ *P. berghei* ANKA (clone 1.49 L) infected red blood cells (iRBCs), as previously described [14]. Parasitemia and mortality were monitored daily. Parasitemia levels were analyzed using Giemsa-stained peripheral blood smears.

### 2.2. Anesthesia and Euthanasia

All efforts were made to prevent undue stress or pain to the mice. Mice with signs of imminent death were euthanized to avoid suffering. Before restraint for X-rays, the mice were given ketamine (100 mg/kg) and xylazine (5 mg/kg). The mice were euthanized with ketamine (300 mg/kg) (Vetbrands, Brazil) and xylazine (22.5 mg/kg) (Syntec, Brazil), and consciousness was checked by testing the pedal reflex, heartbeats and breathing movements.

### 2.3. Histological Evaluations

Necropsy was performed in mice dying naturally from the malaria or mice sacrificed on the 20th days after infection (DAI) to complete the experiment and to avoid animal suffering. The lungs were collected, fixed in buffered 10% formalin and then embedded in paraffin, sectioned at 5 *μ*m onto slides and stained with hematoxylin-eosin (HE) and phosphotungstic acid hematoxylin (PTAH), to emphasize fibrin, as previously described [20].

### 2.4. Arterial Blood Analyses and Measurements of Body Temperature

Mice were placed near a heat lamp for three minutes to increase peripheral blood flow. The mice were then restrained by hand, the ventral artery of the tail was nicked with a small scalpel blade, and capillary tubes containing lithium-heparin (50 IU/mL) were placed underneath the cut to collect approximately 100 *μ*L of blood. The blood was immediately placed in an i-STAT EG 8+ cartridge and analyzed using the* iSTAT* System Analyzer (Abbott group). The PaO_2_/FiO_2_ was calculated assuming that the fraction of inspired O_2_ (FiO_2_) was 0.21. In a subset of mice, the inguinal temperatures were assessed on day 0 and on the 5th, 7th and 9th DAI using a DT-203/60SEC digital thermometer (Becton Dickinson, Franklin Lakes, New Jersey, EUA).

### 2.5. X-Ray

Mice received light anesthesia on the 7th DAI and were X-rayed for 0.066 seconds in a mA100 fine focus Bucky V mAs 6.6 (RAYtech machine KV37, USA). A trained technician blinded to the infection status of the mice examined the X-rays, which were scored for signs of lung injury: 0, no change; 1, discrete and/or light opacification; and 2, diffuse opacification. The mice were later classified as suffering from ALI/ARDS or HP at death by the presence or absence of pleural effusion, respectively.

### 2.6. Determination of Respiratory Pattern

Respiratory patterns (respiratory frequency (RF) and enhanced pause (Penh)) were monitored on the 5th, 7th, 9th, 15th, and 20th DAI by placing the mice in an unrestrained whole-body plethysmography chamber (WBP, Buxco Electronics, Wilmington, North Carolina, USA) for 10 minutes (basal level). The data were collected using Biosystems XA software and included the RF (breaths/minute) and variables to calculate the Penh, a theoretical variable that correlates with both pulmonary resistance and intrapleural pressure [21]. The Penh is calculated by [22]

(1) $$\begin{array}{c} \text{Penh}=\frac{\text{peak}\;\text{expiration}\;\text{speed}}{\text{peak}\;\text{inspiration}\;\text{speed}}\times \left(\frac{\text{expiratory}\;\text{time}}{\text{relaxation}\;\text{time}}-1\right). \end{array}$$

### 2.7. Identifying ALI/ARDS in Mice before Death

To identify ALI/ARDS in mice before death, we used two groups of infected mice: the survival group (infected control) and the sacrificed group in which the mice were sacrificed on the 7th DAI (10–12 mice per group). In the survival group, any mouse showing pleural effusion or red and congested lungs at necropsy, the cause of death was attributed to ALI/ARDS. In contrast, at necropsy, in mice without pleural effusion that died after 13th DAI with pale lungs and high levels of parasitemia, the cause of death was attributed to HP and consequently anemia.

Individual mice sacrificed on the 7th DAI were classified as having been likely to die of ALI/ARDS or HP, by comparing their respiratory patterns and parasitemia levels with the survival group, in which the* causa mortis* was known (Supplementary Figure S1 in Supplementary Material available online at http://dx.doi.org/10.1155/2014/872464).

In each individual experiment, using the survival group, we established three cut-offs using receiver operating characteristic (ROC) curves for the Penh, RF, and parasitemia measured on the 7th DAI, which were used as predictive criteria. The cut-offs from this group were chosen based on the maximum sensitivity and specificity for each parameter. The mice sacrificed on day 7 were also screened for the same parameters before sacrifice, and they were grouped based on the cut-offs from the ROC curves generated using data from the survival group. The same cut-offs were used to retrospectively classify the sacrificed group as suffering from ALI/ARDS or HP. The mice were said to have suffered from ALI/ARDS if they were above the cut-off for at least two of the three variables. For this method to work, it was necessary that three or more animals died by ALI/ARDS in the survival group/per experiment. In all of the experiments, we calculated the sensitivity and specificity from the survival group.

### 2.8. Confirming the Accuracy of the Groupings

To confirm if and when the mice could be grouped using the respiratory pattern cut-offs and parasitemia, confirmation experiments were performed. Two survival groups were assessed for pleural effusion and reddish lungs, which were used as the gold standards for mice dying of lung injury. These criteria constitute a practical phenotype for assessing ALI/ARDS because they are not arbitrary and can be assessed immediately during the necropsy; furthermore, previous results have shown that 100% of the mice that die between 7–12 DAI with clear signs of ALI, including the presence of pulmonary edema, hemorrhages, and hypoxemia [14]. The survivors were monitored until the 20th DAI, and the cause of death was determined. At the end of the experiment, the data from one group were used to generate the ROC curves that were then used to classify the mice in the second group on the 7th DAI; these results were then compared with observations of the pathology from necropsy of the second group (Supplementary Figure S2). Confirmation that the second mouse groupings were likely to be correct was performed in experiments in which the sensitivity and specificity of the groupings using the ROC curves were calculated.

### 2.9. Lung Permeability and Edema

To investigate lung permeability, on the 7th DAI, mice were injected intravenously with 0.2 mL of 1% Evans Blue (Sigma). The mice were sacrificed 45 minutes later, and the lungs were weighed and placed in 2 mL of formamide (Merck) for 48 hours at 37°C. The absorbance of the formamide was then measured at *λ*620 nm. The amount of Evans Blue staining per gram of lung tissue was calculated from a standard curve. The sacrificed mice were classified as suffering from ALI/ARDS by the ROC curves generated from a survival group as described above. The lung permeability of the ALI/ARDS and HP mice was expressed as fold increase in relation to that of the NI mice. To further investigate the presence of edema, in a group of survival mice, the lungs were weighed immediately after natural death, and the wet weights were recorded and compared between ALI/ARDS and HP mice of the same age. The mice were confirmed as suffering from ALI/ARDS or HP at death by the presence or absence of pleural effusion, respectively.

### 2.10. VEGF in Serum

On the 7th DAI, mice were anesthetized, and their serum was collected by cardiac puncture. An ELISA kit (R&D Systems) was used to quantify VEGF levels in the serum according to the manufacturer's instructions. The VEGF level of the ALI/ARDS and HP mice was expressed as fold increase in relation to that of the NI mice. The mice were classified as suffering from ALI/ARDS or HP by comparing the predictive criteria and VEGF levels.

### 2.11. Hematological Parameters Determination

Blood samples were collected in tubes containing sodium citrate as anticoagulant. Total number of red blood cells, hemoglobin, and hematocrit were measured using V-53 reagent kit (Mindray, P.R. China) and Auto Hematology Analyzer BC-5300Vet (Mindray, Nanshan, Shenzhen, P.R. China).

### 2.12. Statistical Analysis

The data were analyzed by D'Agostino-Pearson normality test. Nonparametric variables were compared using Mann-Whitney test. The simultaneous effects of two factors were analyzed by two-way ANOVA following Bonferroni post-hoc test. The differences between the groups were considered significant when *P* ≤ 0.05. Statistical analyses were performed in GraphPad Prism version 5.0, including assessments of sensitivity and specificity. To establish cut off from data, ROC curves were generated using the results of the control group in MedCalc version 8.2.1.0. The Penh, RF and, EP data were analyzed using SPSS for Microsoft version 19.0 through a Pearson correlation (Penh versus RF: −0.827, *P*: 0.001; Penh versus EP: −0.152, *P*: 0.001; RF versus EP: 0.200, *P*: 0.05) for further grouping. The cluster analysis was developed by case grouping using Ward's method with a Euclidean distance analysis, which generated a dendrogram grouping each of the subjects studied and their physiologic characteristics analyzed into a particular cluster.

## 3. Results

### 3.1. Overview of Pathology at Death in* Pb*ANKA-Induced ALI/ARDS

**Figure 1:**
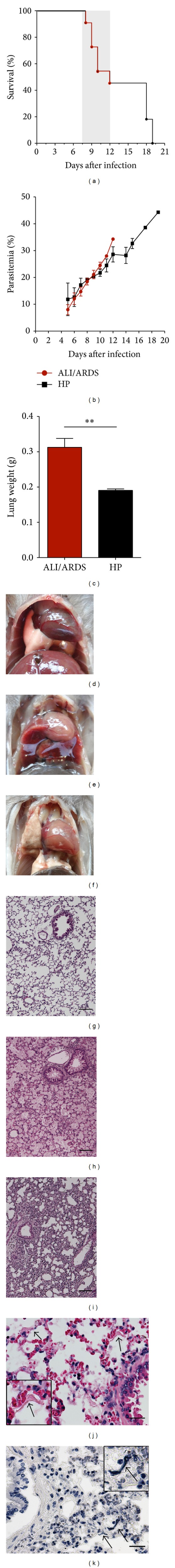
Infection of DBA/2 mice with* P. berghei* ANKA constitutes a rodent model for malaria-associated ALI/ARDS. (a) Survival and (b) parasitemia curves from the ALI/ARDS and HP mice over time. The red line was the mice that died with ALI/ARDS. The gray area represents the period when the mice die of ALI/ARDS. The data presented are representative of 13 independent experiments; *n* = 10–12 mice/experiment. (c) The lungs of mice that died with ALI/ARDS weighed 40% more than the lungs of mice that died with HP (**P ≤ 0.005; Mann-Whitney test of lung weights are representative of four separate experiments). Data ((b) and (c)) represent means and SEM; *n* = 10–12 mice/experiment. (d) Representative pictures of a NI mouse, (e) an infected mouse that died with ALI/ARDS showing hemorrhagic lungs and a large amount of pleural effusion, and (f) a mouse that died with HP showing pale and grayish lungs and no pleural effusion. Representative histopathological images of lungs from (g) NI mice and infected DBA/2 mice that died with (h) ALI/ARDS and (i) HP on the 10th and 21st days after infection, respectively. The arrow points to the hyaline membranes in the lungs of the DBA/2 mice that died with (j) ALI/ARDS stained with hematoxylin-eosin and (k) stained with phosphotungstic acid hematoxylin. The bar corresponds to 100 *μ*m. HP: hyperparasitemia; ALI/ARDS: acute lung injury/acute respiratory distress syndrome.

To characterize and discriminate the pathology associated with ALI/ARDS or HP, DBA/2 mice were infected with* P. berghei* ANKA-infected red blood cells (iRBCs) and followed until their deaths. Subsequently, the animals were necropsied and the* causa mortis* determined. Survival analysis revealed an average of 49.2% (25–75%) of the mice had died of ALI/ARDS between the 7th and 12th DAI, whereas the HP mice died between the 13th and 21st DAI (Figure 1(a)). Although parasitemias were increased in both groups, the HP group had higher levels of parasitemia, at approximately 40–50% on the day of death (Figure 1(b)). Comparing the lung weights, we observed that the mice that died with ALI/ARDS had heavier lungs (averaging 40% more mass) than the mice that died with HP, suggesting edema (Figure 1(c)).

At necropsy, noninfected (NI) mice had light pink lungs, and no liquid inside of the thoracic cavity was observed (Figure 1(d)). However, mice that died with ALI/ARDS had reddish lungs and pleural effusion (Figure 1(e)). In these mice, the histological changes where characterized by marked alveolar edema and hemorrhage, along with neutrophil-dominant inflammatory cellular infiltration, foamy macrophages in the alveolar and interstitial sites, and destruction of the alveolar septa (Figure 1(h)), as previously described [14]. In contrast to the ALI/ARDS mice, the animals that died with HP after the 13th DAI had grayish lungs and a darkened spleen and liver but no pleural effusion (Figure 1(f)) and severe anemia, with decreases in the number of erythrocytes, hemoglobin level, and hematocrit percentage (Supplementary Figure S3). These HP mice had interstitial pneumonia with mononuclear inflammatory cells but at a later time in the infection, diagnosed on the day of death (Figure 1(i)). Interestingly, in the lungs of ALI/ARDS mice we observed the presence of acellular eosinophilic membranes that adhered to the alveolar ducts and walls and hyaline membranes, a hallmark of ALI/ARDS in humans (Figures 1(j) and 1(k)).

### 3.2. Chest Radiography Shows Lung Opacity in* Pb*ANKA-Induced ALI/ARDS

**Figure 2:**
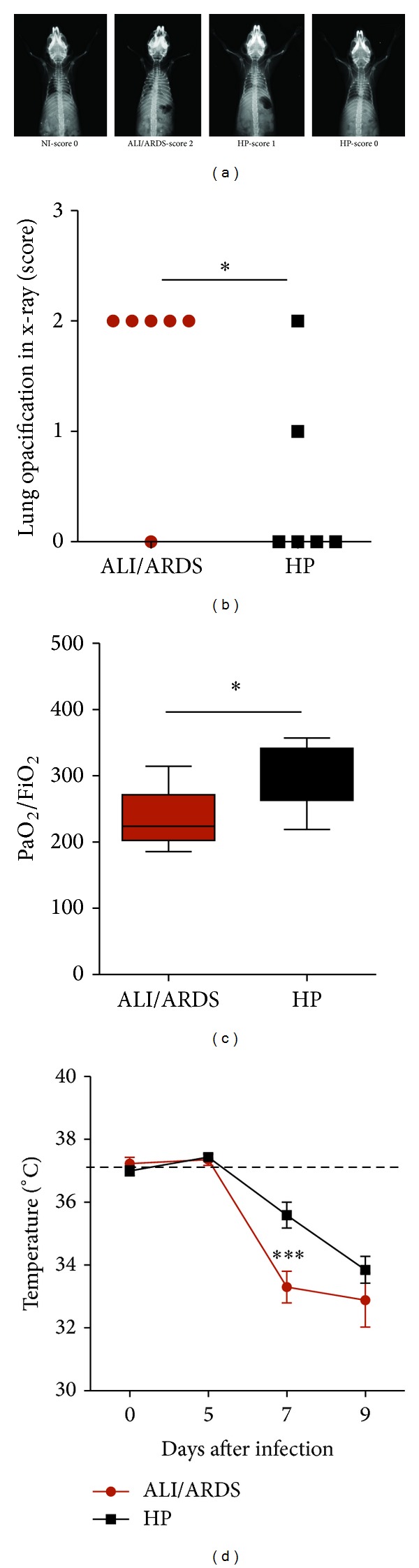
Radiography of the lungs, hypoxemia, and body temperature over time. (a) From left to right, X-rays from NI and infected DBA/2 mice that died with ALI/ARDS and HP showing different lung opacification scores on the 7th DAI. (b) Lung opacification scores on the 7th DAI. Mice that will later die with ALI/ARDS have a higher lung opacification score compared with mice that will die with HP (*n* = 12; *P ≤ 0.05, Mann-Whitney test of scores taken from two separate experiments). NI mice do not have any lung opacification and are assigned score zero. (c) PaO_2_/FiO_2_ values in* P. berghei* ANKA-infected mice on the 7th DAI. Results from three grouped experiments (*n* = 13 mice, *P ≤ 0.05; Mann-Whitney test). (d) Body temperatures in DBA/2 mice infected with* P. berghei* ANKA slightly increased on the 5th DAI from 37.1°C in the NI mice to 37.3 in the ALI/ARDS mice and 37.4°C in the HP mice). However, the temperatures dropped and the mice became hypothermic (especially the ALI/ARDS mice), with mean temperatures of 33.0°C on the 7th DAI and 32.8°C on the 9th DAI. Results are from three grouped experiments (*n* = 31 mice; ***P ≤ 0.001, two-way ANOVA with Bonferroni post test). Data (d) represents means and SEM. The dashed line represents the mean value of NI mice. NI: noninfected mice; ALI/ARDS: acute lung injury/acute respiratory distress syndrome; HP: hyperparasitemia.

Bilateral infiltrates observed on frontal chest radiographs are recognized as a criterion for the diagnosis of ALI and ARDS [23]. In our work, X-ray analysis on the 7th DAI revealed lung opacification, which was more prominent in the ALI/ARDS group than in the HP group (Figures 2(a) and 2(b)). NI mice had no changes in the lungs.

### 3.3. *Pb*ANKA-Induced ALI/ARDS Is Associated with Hypoxemia and Decreased Body Temperature

Hypoxemia is not a direct assessment of damage* per se* but is often a manifestation of injury [24]. In humans, the hypoxemia was defined by The American-European Consensus Conference as PaO_2_/FiO_2_ ≤ 300 mmHg (for ALI) or ≤200 mmHg (for ARDS) [25]. In agreement with previous results [14], we show that the majority of the DBA/2 mice infected with* Pb*ANKA who died of ALI/ARDS had PaO_2_/FiO_2_ values between 200 and 300 mmHg, and we further demonstrate that mice that developed the more severe form of ARDS, presented PaO_2_/FiO_2_ values of ≤200 mmHg. On the 7th DAI, the PaO_2_/FiO_2_ in the ALI/ARDS group (234.3 ± 21.38) was significantly lower than the level in the HP group (303 ± 17.26; *P* = 0.029) (Figure 2(c)). NI mice showed PaO_2_/FiO_2_ values above 300 mmHg with average 371.42 (SD ± 24.27). There are no agreed-upon validated PaO_2_/FiO_2_ data in animal models of lung injury [26]; thus, we categorized all of the animals in the group with lung injury as ALI/ARDS.

The infected mice had slightly increased body temperatures between day 0 and the 5th DAI; however, between the 7th and 9th DAI, their temperatures dropped and the mice became hypothermic (Figure 2(d)). Mice that would start to develop ALI/ARDS and die had the lowest temperatures on the 7th DAI compared with the HP mice. We hypothesized that the reduction in body temperature could be related to decreased survival of the animals, especially those that developed ALI/ARDS.

### 3.4. Respiratory Patterns and Parasitemia Are Correlated in* Pb*ANKA-Induced ALI/ARDS

**Figure 3:**
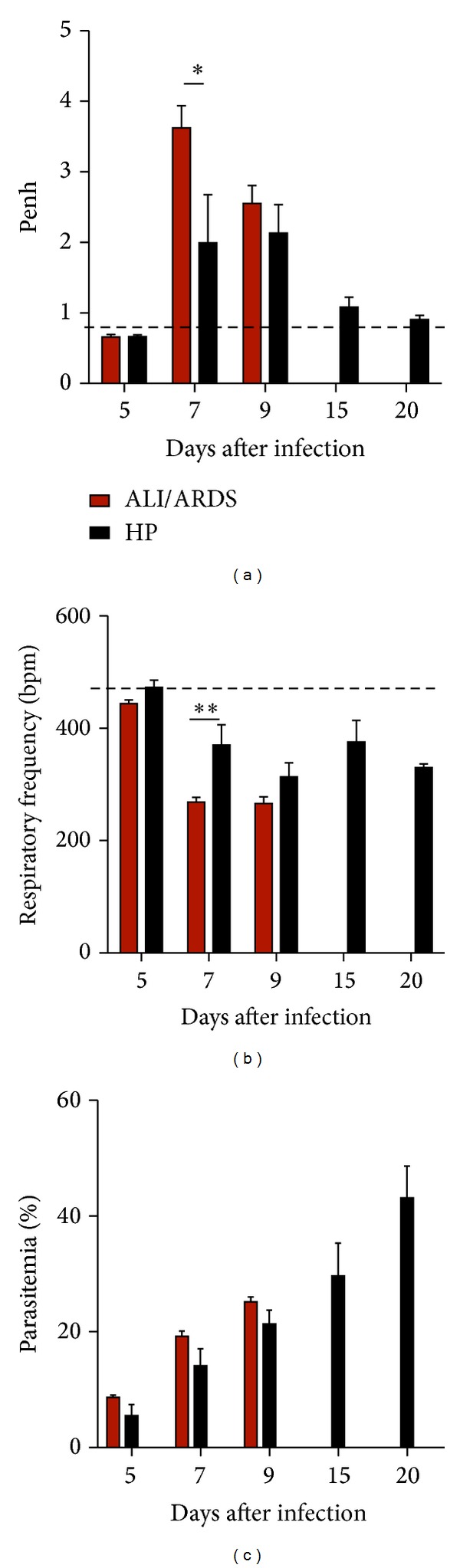
Breathing patterns and parasitemia from ALI/ARDS and HP mice over time. (a) and (b) Breathing patterns and (c) parasitemia from DBA/2 mice infected with* P. berghei* ANKA that developed ALI/ARDS and HP over time. (a) There was no evidence on the 5th and 9th DAI that the ALI/ARDS and HP mice had different breathing patterns. However, on the 7th DAI, there was evidence that the ALI/ARDS mice had a higher enhanced pause (Penh) (a) and a lower respiratory frequency (b) than the HP mice. Parasitemia increased over time in both groups (c). Results are representative from three independent experiments (*n* = 11 mice/experiment; *P ≤ 0.05, two way ANOVA with Bonferroni post test). The dashed line represents the mean value of NI mice; NI: noninfected mice; ALI/ARDS: acute lung injury/acute respiratory distress syndrome; HP: hyperparasitemia.

To characterize the lung physiopathology during infection, we analyzed the enhanced pause (Penh), respiratory frequency (RF), and parasitemia (EP) levels at five different time points. On the 5th DAI the ALI/ARDS group had breathing patterns and parasitemia similar to the HP group and NI mice. However, by the 7th DAI, the ALI/ARDS group had increased Penh, decreased RF, and a tendency to increase the parasitemia even if not significantly, compared with the HP mice (Figures 3(a)–3(c)). After the 9th DAI, the statistical comparison between the ALI/ARDS and HP mice could not be performed in the individual experiments due to the minimal numbers of surviving ALI/ARDS mice. Interestingly, animals that survived for longer periods, that is, animals that did not develop ALI/ARDS and subsequently died by HP, breathing patterns returned nearly to baseline levels. NI mice had no changes in breathing patterns over time.

Parasitemia increased over the course of infection (Figure 3(c)). On the day of death, parasitemia in the ALI/ARDS group was 20.8% (SD ± 4.6), while parasitemia in mice that died of HP was 40.9% (SD ± 9.21; *P* ≤ 0.0001). However, on the 7th DAI, the mice that went on to die of ALI/ARDS had 17.0% (SD ± 5.0) iRBCs, while the mice that would go on to die with HP were 12.2% (SD ± 4.5) parasitemic (Supplementary Figure S4a).

Aiming to perform a correlation study examining the respiratory parameters (Supplementary Figure S4b and S4c) and EP (Supplementary Figure S4a), we conducted experiments on the 7th DAI, when the onset of important pulmonary pathology occurred, rather than using the 5th DAI when we did not observe consistent differences in respiratory patterns between the study groups.

**Figure 4:**
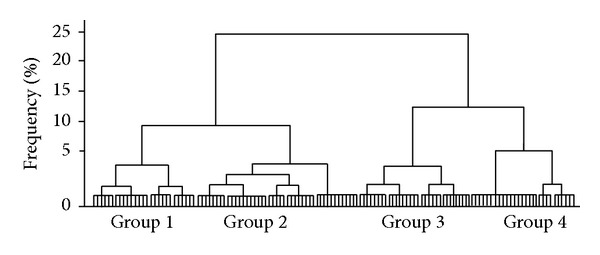
Breathing patterns and parasitemia could be used to group mice into two main clusters. Ward's linkage cluster analysis illustrates the distance between the physiological cluster patterns in DBA/2 mice infected with* P. berghei* ANKA that developed ALI/ARDS and HP. Values were measured on the 7th DAI. The data are from 13 independent experiments; *n* = 142 mice. Group 1 = 88.46% of individuals with ALI/ARDS and 11.53% with HP; group 2 = 57.77% of individuals with ALI/ARDS and 42.22% with HP; group 3 = 19.35% individuals with ALI/ARDS and 80.64% with HP; group 4 = 21.42% of individuals with ALI/ARDS and 78.57% with HP. ALI/ARDS: acute lung injury/acute respiratory distress syndrome; HP: hyperparasitemia.

The high correlation between Penh, RF, and EP and the development of ALI/ARDS or HP (Pearson correlation Penh* versus* RF: −0.827, *P* = 0.001; Penh* versus* EP: −0.152, *P* = 0.001; RF* versus* EP: 0.200, *P*: 0.05) led us to identify four groups based on the cluster analysis (group 1 consisted of 88.46% of individuals with ALI/ARDS and 11.53% with HP; group 2 included 57.77% of individuals with ALI/ARDS and 42.22% with HP; group 3 consisted of 19.35% individuals with ALI/ARDS and 80.64% with HP; group 4 included 21.42% of individuals with ALI/ARDS and 78.57% with HP) (Figure 4). Such clustering highlights that the physiological conditions evaluated in groups 1 and 2 were dominated by ALI/ARDS, while groups 3 and 4 were dominated by HP, with a difference of 25%. Additionally between the groups with major higher frequencies of one of the two pathologies (groups 1 and 2 or groups 3 and 4), the difference between the analyzed characteristics was 10%.

### 3.5. A Murine Model to Predict Malaria-Associated ALI/ARDS at an Early Time Point

**Figure 5:**
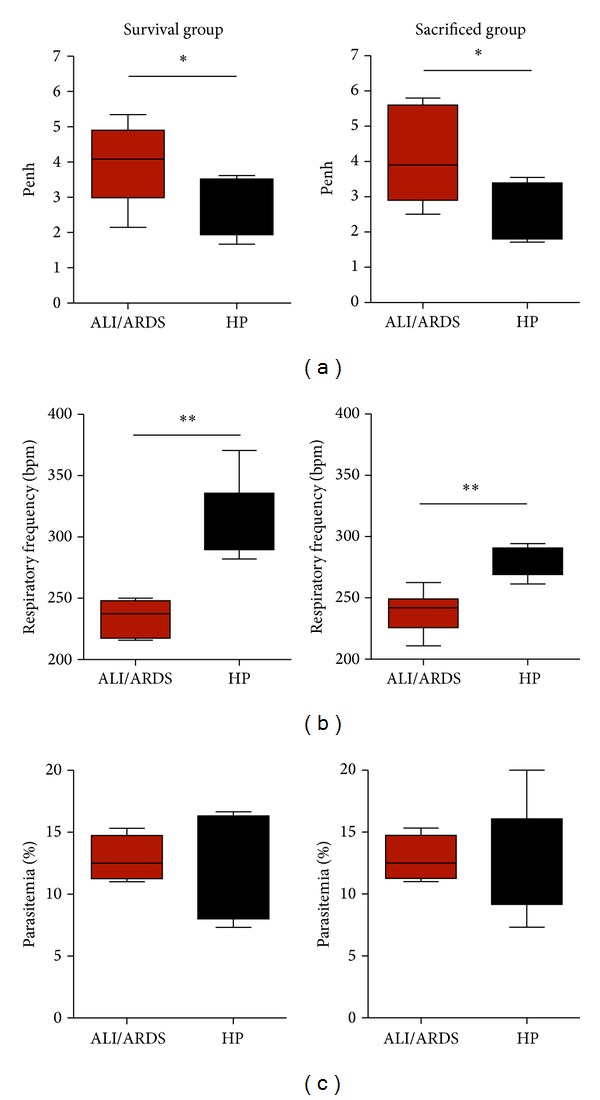
A murine model to predict malaria-associated ALI/ARDS. (a) Penh (enhanced pause), (b) respiratory frequency, and (c) parasitemia measured on the 7th DAI. The sacrificed mice were classified according predictive model, using the parameter cut-offs measured from the survival mice and applied to the sacrificed mice. DBA/2 mice infected with* P. berghei* ANKA and their breathing parameters were measured in plethysmograph chambers (BUXCO Electronics, USA). Note that these three parameters are similar between the survival group and sacrificed group; (*n* = 11 mice/group; *P < 0.05, **P < 0.005, Mann-Whitney test). ALI/ARDS: acute lung injury/acute respiratory distress syndrome; HP: hyperparasitemia; bpm: beats per minute. Results are representative of more than 5 independent experiments.

Mice that die from ALI/ARDS present altered Penh, RF, and EP values from the mice that die from HP; thus, we hypothesized that those parameters could be used as predictive criteria for the* causa mortis*. As described in the materials and methods, for each individual experiment, we used an infected control group (survival group) and established cut-offs using ROC curves for Penh, RF, and EP (Supplementary Table S1) measured on the 7th DAI and applied these cut-offs to classify the sacrifice group (Supplementary Figure S1). Following this procedure, the mice sacrificed on the 7th DAI could be classified as likely to die with ALI/ARDS or HP. We compared their respiratory patterns and parasitemia with a survival group that was not sacrificed and for which the cause of death was known. The sensitivity (≤100% and ≥67%; average 88.31%; SD ± 11.95) and specificity (100% ≤ and ≥71%; average 90.85%; SD ± 10.81) were calculated from the survival group for each individual experiment. In addition, we observed that the respiratory patterns and parasitemia were similar between the survival group and the sacrificed group (Figures 5(a)–5(c)).

To confirm the accuracy of the grouping, the cut-offs from the ROC curves of the respiratory patterns and parasitemia were performed using the two infected control groups (survival groups) on the 7th DAI. The groups were monitored until the 20th DAI, and the cause of death was determined (Supplementary Figure S2). On the 7th DAI, we were able to group the mice in the ALI/ARDS or HP groups with 91% sensitivity and 76% specificity in the three grouped experiments (Table 1). Among the experiments, the best result was 100% sensitivity and 100% specificity, and the worst result was 66.6% sensitivity and 60% specificity. On the 9th DAI, we were not able to group the mice, as no ALI/ARDS mice from the survival group were alive.

### 3.6. *Pb*ANKA-Induced ALI/ARDS Causes Breakdown of the Alveolar-Capillary Barrier

**Figure 6:**
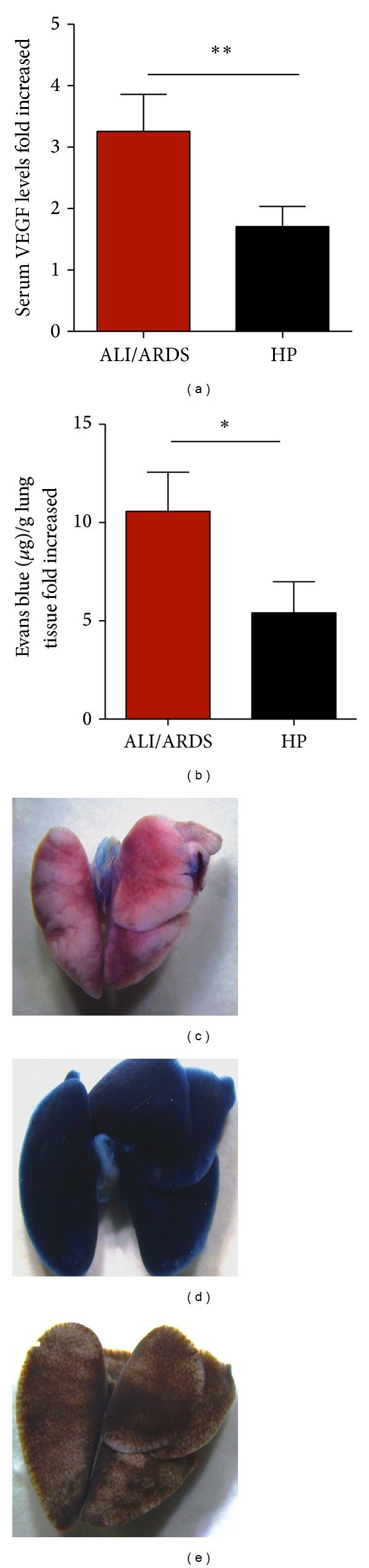
Increased vascular permeability and serum VEGF protein confirmed the predictive criteria for malaria-associated ALI/ARDS. (a) Serum VEGF protein in DBA/2 mice infected with* P. berghei* ANKA on the 7th DAI was measured by ELISA. The VEGF levels are higher in the mice classified as likely to die with ALI/ARDS compared with HP mice (according to the proposed predictive criteria). The data represent fold increases in relation to NI mice taken from three experiments; (*n* = 28 mice; **P < 0.005, Mann-Whitney test). (b) Lung vascular permeability in DBA/2 mice infected with* P. berghei* ANKA 7th DAI, assessed using Evans Blue. The vascular permeability is higher in the mice classified as likely to die with ALI/ARDS compared with the HP mice (according to the proposed predictive criteria). The data represent fold increased in relation to NI mice taken from three experiments; (*n* = 51 mice; *P < 0.05, Mann-Whitney test). Bars represent means and SEM. The images represent (c) NI: noninfected mice, (d) ALI/ARDS: acute lung injury acute respiratory distress syndrome, and (e) HP: hyperparasitemia.

In a previous study, we demonstrated that VEGF promotes malaria-associated ALI in mice and that expression of this growth factor is increased in mice that died of ALI [14]. Here, using blood samples from mice sacrificed on the 7th DAI, we confirmed that mice classified as likely to die with ALI/ARDS and HP had VEGF serum levels 3.3-fold and 1.7-fold higher than those in the NI group, respectively (Figure 6(a)). Furthermore, the pulmonary vascular permeability measured by Evans blue uptake in the lungs on the 7th DAI was higher in mice predicted to die of ALI/ARDS (10.6-fold higher than NI mice) than in those classified as suffering from HP (5.5-fold higher than NI mice) (Figures 6(b)–6(e)).

## 4. Discussion

According to The American-European Consensus Conference, the recommended criteria to define both ALI and ARDS are acute onset, hypoxemia levels of PaO_2_/FiO_2_ ≤ 300 mmHg (for ALI) or ≤200 mmHg (for ARDS), bilateral infiltrates seen on a frontal chest radiograph, and pulmonary artery wedge pressure (≤18 mmHg when measured or no clinical evidence of left atrial hypertension) (revised by Thompson and Moss, 2010). Recently, in accord with The Berlin Definition, the ALI term was abolished and the ARDS was defined based on oxygenation: mild ARDS (200 mmHg PaO_2_/FiO_2_ ≤ 300 mmHg), moderate ARDS (100 mmHg PaO_2_/FiO_2_ ≤ 200 mmHg), and severe ARDS (bellow 100 mmHg PaO_2_/FiO_2_, among other criteria [27]. However, The Berlin Definition was not validated by a later study [28] and ALI is still used for lung injury in mice. Despite the discussion in the field, some consensual parameters are used to study ALI in mice such as kinetics of injury, radiographic evaluation, physiological assessment, histological evidence of lung injury, and assessment of increased permeability of the alveolar-capillary membrane [26].

Malaria-associated ALI/ARDS is progressively more frequently reported, is often fatal and is still not fully understood [29], thus an animal model would be essential to address such a complex disease [30]. We showed that DBA/2 mice infected with* Pb*ANKA constitute a rodent model of malaria-associated ALI/ARDS [14], and here, we approach respiratory and parasitological parameters to devise a mathematical model able to predict development of ALI/ARDS during * Pb*ANKA infection.

This murine model clearly showed edema in ALI/ARDS mice, indicating that these animals had more severe disease and that they were dying during the exudative phase of ALI/ARDS. In addition, we observed local destruction of the alveolar epithelium, with denuded areas covered by fibrin-containing hyaline membranes, a hallmark of ALI/ARDS in humans [23], along with neutrophils, alveolar macrophages, mononuclear cells, and iRBC. The presence of edema observed in the ALI/ARDS mice is also reflected by the increased wet weight of the lungs [31], although the increased weight of the lungs could be partially due to cellular accumulation [18]. The opaque appearance of the lungs in X-rays and the opaque pulmonary alveolar pattern were sometimes bilateral and other times unilateral. However, we did not observe type II cell hyperplasia in the ALI/ARDS mice or the interstitial fibrosis characteristic of the proliferative phase of ARDS, denoting the early stage of the acute syndrome.

Pleural effusion was used as a gold standard for mice dying of lung injury, as it is a practical nonarbitrary phenotype to assess. Even though this finding is infrequent in human malaria, pleural effusion has been observed in malaria-associated ARDS in humans and in a nonmalarial setting and has previously been correlated with reduced gas exchange in the lungs [32–34].

Here, we showed that respiratory patterns and parasitemia differ between the mice that would go on to die with ALI/ARDS or HP in a model of malaria-associated lung injury. We further show that we could use these differences to accurately group the mice as likely to die with ALI/ARDS as early as the 7th DAI but not on the 5th DAI. On the 7th DAI, there were significant differences in these parameters between the two groups, and the use of a cut-off from the ROC curves enabled us to identify the mice that would die with ALI/ARDS, with 88.5% (SD ± 11.52) sensitivity and 90.7% (SD ± 11.45) specificity at this point using the cut-off for Penh, RF, and EP from the survival group as a template for the sacrificed group.

The ability to identify mice as being likely to die of ALI/ARDS accurately based on respiratory patterns and parasitemia opens up the possibility of studying this disease without concerns for confounding data generated from mice that would never go on to develop the disease or having to wait until death is imminent. Altered respiratory patterns are associated with lung injury and have been observed in a number of respiratory models, including Penh data that have been used in different murine models [35–37]. The high parasitemia that is associated with adverse outcomes has been shown in a number of murine malaria models [38, 39], further supporting our choice to use these parameters to distinguish mice suffering from ALI/ARDS. Despite some controversy regarding the use of Penh [20], our results clearly show that this parameter varies between mice that will or will not die of ALI/ARDS.

ARDS in humans causes tachypnea [23]. Nevertheless, the current data showed the ALI/ARDS mice had a lower RF than the HP mice. In addition, our results showed that animals with ALI/ARDS experienced a sharp decline in body temperature, especially on the 7th DAI. Despite malaria being traditionally known as a febrile illness [40, 41], murine malaria, including ARDS, can lead to hypothermia [18, 42, 43]. This symptom may be an interesting effort to reduce inflammation-mediated damage to the endothelium, as it has been shown that increased temperatures result in increased sensitivity of endothelial cells to proinflammatory factors such as tumor necrosis factor [44]. We suggest that decreased RF may be a side effect of hypothermia; it may also be associated with the increased effort required by the mice to breathe due to the lung damage, edema and/or hypothermia that may have contributed to the development of ALI/ARDS and the death of these animals.

Even though the parasitemia average was higher in the ALI/ARDS group on the 7th DAI in the 13 experiments observed (Supplementary Figure S4a), it was the most variable analyzed parameter. In our predictive model, even small differences often helped to define whether an animal would be classified with ALI/ARDS or HP because the proposed method combines two or three parameters at the same time. The high correlation identified between the Penh, RF, and EP and the development of ALI/ARDS or HP exhibited in the development of these pathologies by cluster analysis (Figure 4) with any differentiation factors studied under 5% could allow a positive identification of ALI/ARDS with an accuracy varying from 57.77% to 88.46% or of HP with an accuracy varying from 78.57% to 80.64%. Furthermore, these experiments revealed a large difference in these variables between the groups ranging from 25% to 10% in terms of physiological conditions studied, enabling us to establish parameters to predict the presence or absence of ALI/ARDS or HP with greater accuracy in our study model either with a refinement of the data or with the inclusion of one or more variables.

Previously, it was shown that lung vessel permeability and VEGF levels were significantly higher in infected DBA/2 mice exhibiting ALI symptoms when death is imminent [14]. Here, we confirmed similar these results using this new predictive criteria to classify these mice.

How* Plasmodium* infection causes ALI/ARDS remains largely unknown. Animal models have the potential to elucidate the mechanisms of disease and identify prognostic markers and therapeutic targets. The results presented in this paper describe a murine model of ALI/ARDS and, most importantly, describe how it is possible to accurately identify mice with lung injury before death. The study of mechanisms involved in the genesis of ALI/ARDS on earlier time points is essential for the elucidation of the pathogenic events underlying the development of this severe disease.

## Supplementary Material

The Supplementary Figure S1 shows the method followed to identify ALI/ARDS mice before death by using predictive criteria (Penh, respiratory frequency and parasitemia). Supplementary Figure S2 shows the accuracy of the applied method. The Supplementary Figure S3 confirms that *P. berghei* ANKA infection leads to severe anemia in hyperparasitemic mice. The Supplementary Figure S4 demonstrates parasitemia and breathing patterns (Penh, respiratoy frequency) of ALI/ARDS and HP mice on the 7th days after infection. Data represent 13 different experiments. The Supplementary Table S1 shows the cut-offs using the ROC curves for Penh, respiratory frequency and parasitemia in the survival group on the 7th days after infection. The sacrifice group was classified based on the parameters described.

## Figures and Tables

**Table 1:** Confirming the accuracy of the groupings. True and false pathologies checked by the ROC curves from the predictive criteria∗ on the 7th DAI and the *causa mortis*.

Pathology			
Test	ALI/ARDS	HP	Total
Hits	11	13	24
Errors	1	4	5

Total	12	17	29

*Penh, respiratory frequency, and parasitemia.
