# Supplementary material for: Predictive Criteria to Study the Pathogenesis of Malaria-Associated ALI/ARDS in Mice
Source: Mediators Inflamm. 2014 Sep 2;2014:872464. doi: 10.1155/2014/872464 (PMC4167651; doi:10.1155/2014/872464)

Supplementary Table S1. Cut-offs using the ROC curves for Penh, respiratory frequency and parasitemia from 13 experiments measured on the 7th days after infection from the survival group. These data were used to classify the sacrifice group.

Experiments	ROC CURVES OF SURVIVAL GROUP									Attributes of survival groups after parameters associations	
	Penh			Respiratory Frequency			Parasitemia				
	Cut-off (>)	Sensitivity (%)	Specificity (%)	Cut-off (<=)	Sensitivity (%)	Specificity (%)	Cut-off (>)	Sensitivity (%)	Specificity (%)	Sensitivity (%)	Specificity (%)
1	1,87	83	100	252,20	67	100	10,66	100	50	83	100
2	3,25	75	100	345,80	75	75	13,66	100	75	100	100
3	3,38	100	50	268,90	100	75	12,66	100	75	75	71
4	3,62	67	100	250,00	100	90	12,66	67	40	100	90
5	2,4	80	63	259,20	80	100	13,33	60	75	67	90
6	1,02	100	86	195,70	100	100	12,66	100	100	100	100
7	1,77	100	71	325,00	67	100	15,33	67	86	100	75
8	2,42	75	100	312,20	100	100	17,00	100	100	80	100
9	2,38	80	80	276,00	80	60	13,33	100	100	80	100
10	3,66	40	100	306,90	80	100	13,60	100	75	80	90
11	3,46	100	100	243,00	100	100	20,10	75	100	100	100
12	2,69	100	75	254,50	80	100	11,50	80	75	83	75
13	2,83	83	80	290,50	100	80	17,66	80	80	100	90
**Range**	**1.022-3.664**	**40-100**	**50-100**	**195.7-345.8**	**67-100**	**60-100**	**10.66-20.1**	**60-100**	**40-100**	**67-100**	**71-100**
**Average**	**2,67**	**83,33**	**85,00**	**275,38**	**86,8**	**90,77**	**14,17**	**86,8**	**79,28**	**88,31**	**90,85**
**SD**	**0,80**	**17,55**	**16,85**	**40,12**	**13,47**	**13,67**	**2,66**	**15,75**	**18,82**	**11,95**	**10,81**

Supplementary Figure S1

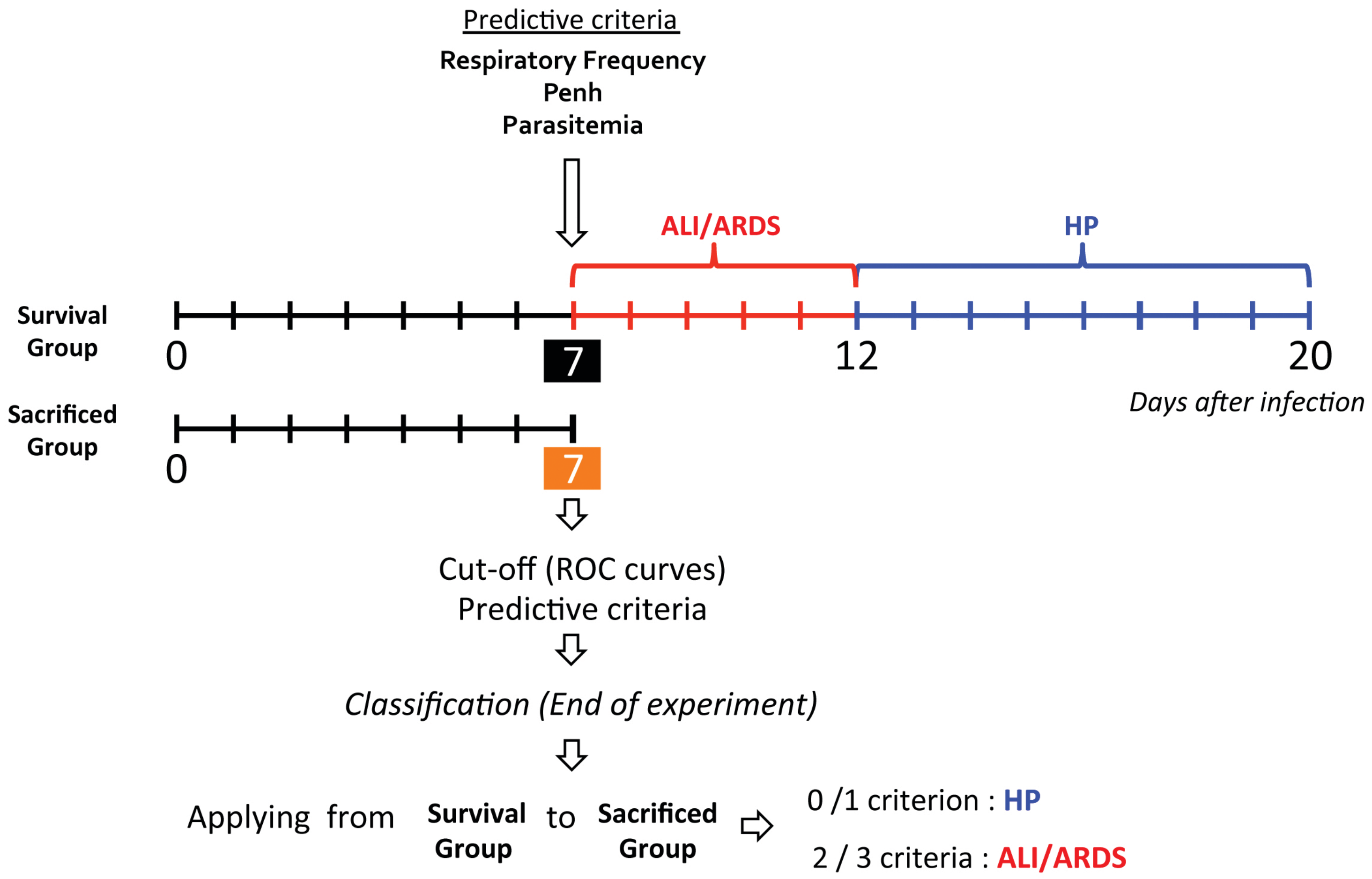

Supplementary Figure S2

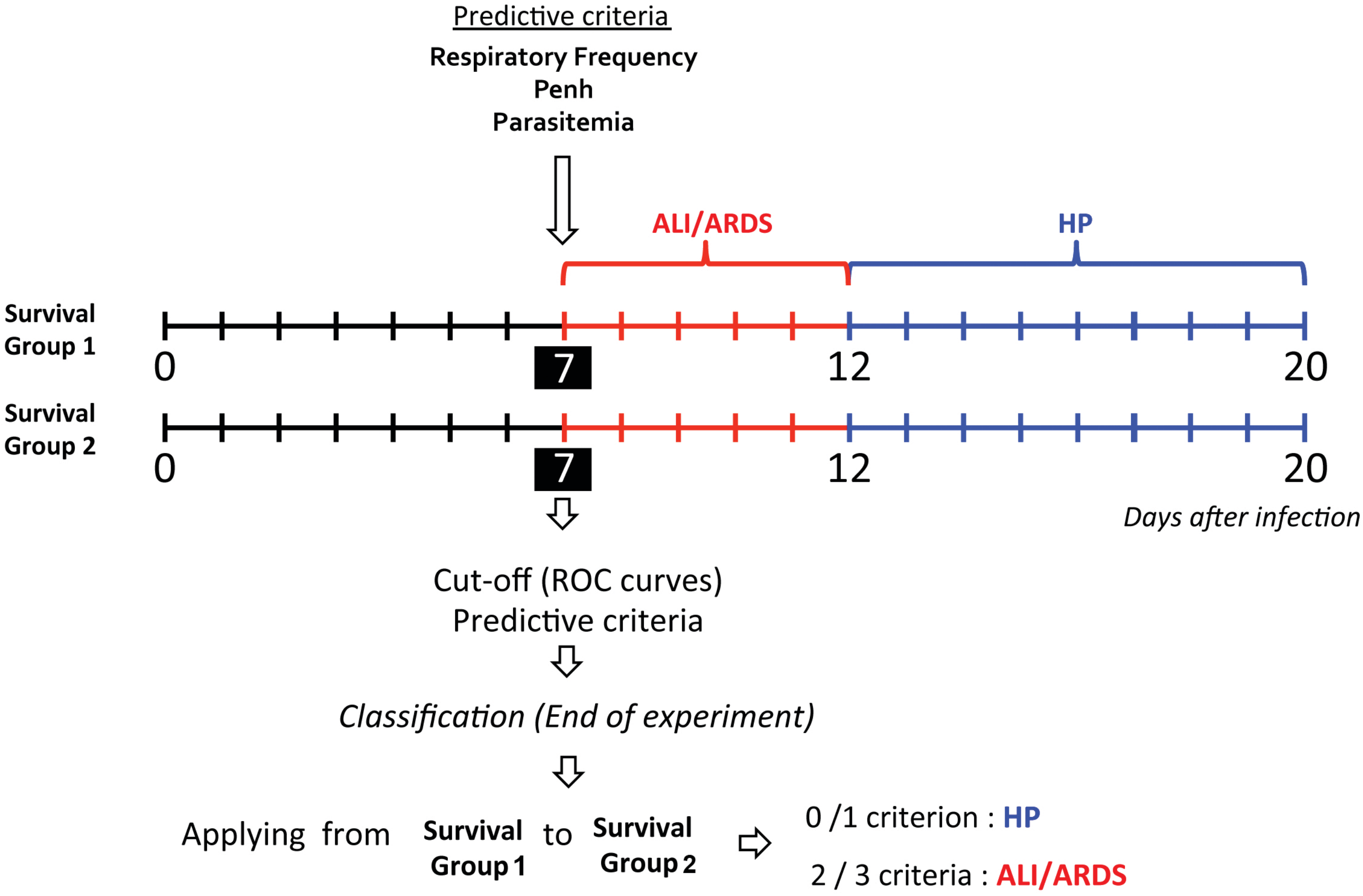

Supplementary Figure S3

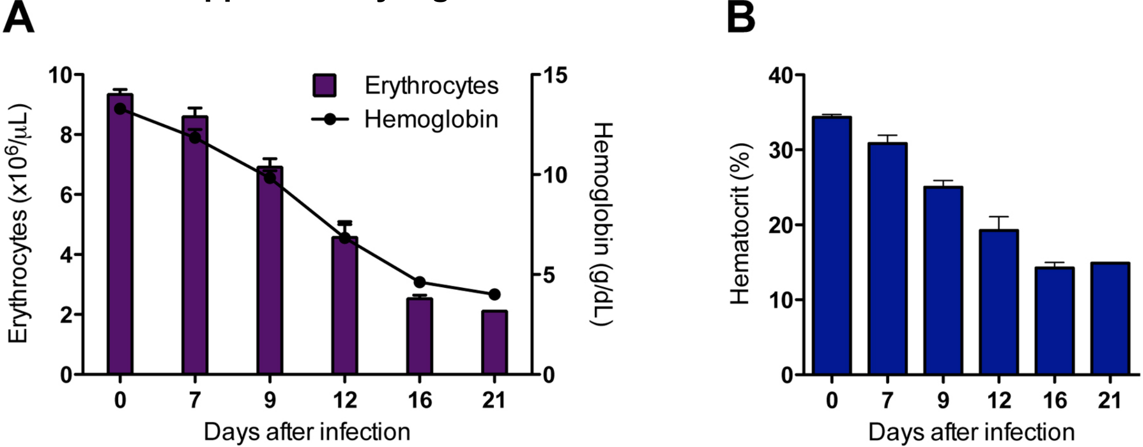

Supplementary Figure S4

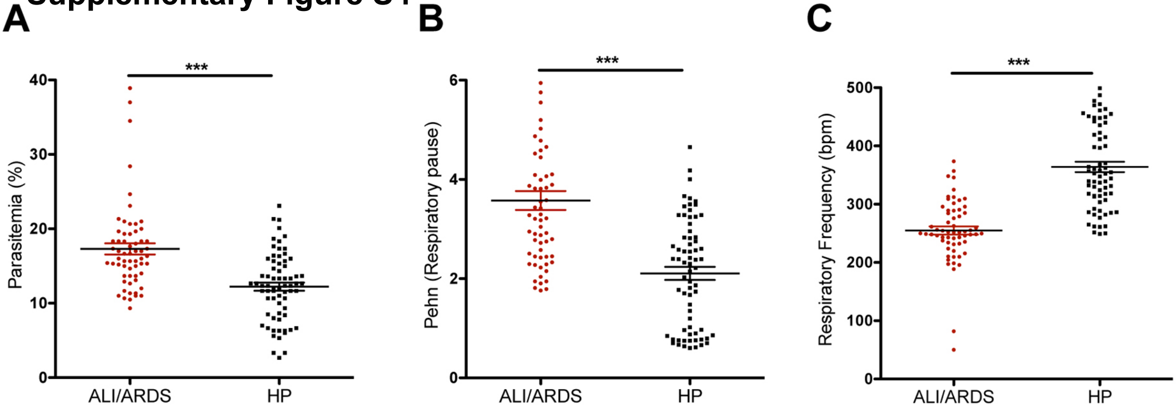

Supplement: Supplementary file 1 — The Supplementary Figure S1 shows the method followed to identify ALI/ARDS mice before death by using predictive criteria (Penh, respiratory frequency and parasitemia). Supplementary Figure S2 shows the accuracy of the applied method. The Supplementary Figure S3 confirms that P. berghei ANKA infection leads to severe anemia in hyperparasitemic mice. The Supplementary Figure S4 demonstrates parasitemia and breathing patterns (Penh, respiratoy frequency) of ALI/ARDS and HP mice on the 7th days after infection. Data represent 13 different experiments. The Supplementary Table S1 shows the cut-offs using the ROC curves for Penh, respiratory frequency and parasitemia in the survival group on the 7th days after infection. The sacrifice group was classified based on the parameters described. [file 872464.f1.pdf]
